# The Intriguing Regulators of Muscle Mass in Sarcopenia and Muscular Dystrophy

**DOI:** 10.3389/fnagi.2014.00230

**Published:** 2014-08-29

**Authors:** Kunihiro Sakuma, Wataru Aoi, Akihiko Yamaguchi

**Affiliations:** ^1^Research Center for Physical Fitness, Sports and Health, Toyohashi University of Technology, Toyohashi, Japan; ^2^Laboratory of Health Science, Graduate School of Life and Environmental Sciences, Kyoto Prefectural University, Kyoto, Japan; ^3^Department of Physical Therapy, Health Sciences University of Hokkaido, Kanazawa, Japan

**Keywords:** sarcopenia, muscular dystrophies, autophagy, myostatin, serum response factor, mTOR

## Abstract

Recent advances in our understanding of the biology of muscle have led to new interest in the pharmacological treatment of muscle wasting. Loss of muscle mass and increased intramuscular fibrosis occur in both sarcopenia and muscular dystrophy. Several regulators (mammalian target of rapamycin, serum response factor, atrogin-1, myostatin, etc.) seem to modulate protein synthesis and degradation or transcription of muscle-specific genes during both sarcopenia and muscular dystrophy. This review provides an overview of the adaptive changes in several regulators of muscle mass in both sarcopenia and muscular dystrophy.

## Introduction

In humans, skeletal muscle is the most abundant tissue in the body, comprising 40–50% of body mass and playing vital roles in locomotion, heat production during periods of cold stress, and overall metabolism. Skeletal muscle is composed of bundles of muscle fibers called fascicles. The cell membrane surrounding the muscle cell is the sarcolemma, beneath which lies the sarcoplasm, which contains the cellular proteins, organelles, and myofibrils: the titin actin filament and the thicker myosin filament. The arrangement of these protein filaments gives skeletal muscle its striated appearance. Skeletal muscle is capable of remarkable adaptations in response to altered activity. These adjustments to mechanical and metabolic demands elicit marked modifications of gene expression that could lead to gain (hypertrophy) or loss (atrophy) of muscle mass. Whereas, endurance training leads to minor changes in skeletal muscle mass, strength training induces marked hypertrophy of exercising muscles. Resistance training [full squat, leg press, and leg-extension, three sets to failure of 6–8 RM (~80–85% of the 1 RM, Monday) and 10–12 RM (~70–75% of the 1 RM, Friday), 18 weeks] for young sedentary subjects (women, 21.4 ± 1.4-year old) elicited a 10–30% increase in fiber cross-sectional area of the vastus lateralis muscle (Staron et al., 1991).

Loss of muscle is a serious consequence of many chronic diseases and of aging itself because it leads to weakness, loss of independence, and increased risk of death. Unfortunately, the field suffers from having more definitions than therapies; muscle wasting is an inevitable part of aging, where it is known as sarcopenia (Rosenberg, 1989). Muscle loss is also common in muscular dystrophy, in which markedly loss of various membranous structural proteins occurs around muscle fibers (Vainzof et al., 2008). Intriguingly, sarcopenia and muscular dystrophy possess similar characteristics, including the accumulation of fibrosis, a wide-range fiber size distribution, and central nuclei (Sakuma et al., 2008; Vainzof et al., 2008; Berger and Doherty, 2010; Hepple, 2012).

In hypertrophied muscle, increasing protein synthesis and decreasing protein degradation are also important events. Phosphatidylinositol-3-kinase (PI3-K)/Akt/mammalian target of rapamycin (mTOR) signaling has been shown to be crucial to protein synthesis (Glass, 2010; Sakuma and Yamaguchi, 2012b). Mechanical stretching *in vivo* and *in vitro* activates serum response factor (SRF)-dependent signaling in skeletal muscle (Gauthier-Rouviére et al., 1996; Sakuma and Yamaguchi, 2012a). In contrast, negative regulators are proposed to induce muscle atrophy by inhibiting protein synthesis and enhancing protein degradation in skeletal muscle. For example, the ubiquitin–proteasome system (UPS) is thought to be a major contributor for degrading many structural proteins (Cao et al., 2005). However, the autophagy–lysosome system has been largely ignored despite evidence that lysosomal degradation contributes to protein breakdown in atrophying muscles (Furuno et al., 1990). Sandri (2010, 2011) has shown that the autophagy–lysosome and UPS are coordinately regulated during muscle wasting. On the other hand, myostatin is a potent inhibitor of muscle growth and is considered as a therapeutic target for muscle wasting including cachexia and sarcopenia, muscular dystrophy, and amyotrophic lateral sclerosis (Sakuma and Yamaguchi, 2011b).

Several positive and negative regulators (mTOR, SRF, atrogin-1, p62, and myostatin) have been proposed to enhance protein degradation or transcription of muscle-specific genes during both sarcopenia and muscular dystrophy. However, the adaptations of these important mediators were not necessarily similar in these two conditions. Muscle ring finger 1 (MuRF-1), an E3 ubiquitin ligase, is activated in many different types of muscular dystrophy (Saenz et al., 2008; Fanin et al., 2013, 2014), but many mediators of UPS do not change during sarcopenia (Sakuma et al., 2014). Several studies have indicated similar dysfunctions of autophagic signaling during sarcopenia and muscular dystrophy (De Palma et al., 2012; Sakuma et al., 2014). In addition, skeletal muscle in both conditions exhibits down-regulation of SRF (Sakuma et al., 2004, 2008) and appears to show the activation of myostatin-dependent signaling (Sakuma et al., 2004; McKay et al., 2012). In contrast, the adaptation of mTOR-dependent signaling seems to differ between sarcopenia and muscular dystrophy to some extent (De Palma et al., 2012; Sakuma et al., 2014). To build on these previous findings, more descriptive and comprehensive comparison of positive and negative muscle regulators between sarcopenia and muscular dystrophy is needed.

Therefore, in this review, we concentrate on specific alterations discussed in the recent literature that are present in the skeletal muscle in both muscle wasting disorders. In addition, we focus on the adaptive changes in positive and negative regulators (mTOR, UPS, autophagy, etc.) of muscle mass. If we can understand more concretely and definitively the mechanisms underlying sarcopenia and muscular dystrophy, more effective applications (nutritional and/or pharmacological) for skeletal muscle wasting may be conducted in the near future.

## Characteristics of Sarcopenia and Muscular Dystrophy

### Sarcopenia

Aging is associated with a progressive decline of muscle mass, quality, and strength, a condition known as sarcopenia (Candow and Chilibeck, 2005). Although this term is applied clinically to denote loss of muscle mass, it is often used to describe both a set of cellular processes (denervation, mitochondrial dysfunction, inflammation, and hormonal changes) and a set of outcomes such as decreased muscle strength, mobility, and function (Melton et al., 2000), a greater risk of falls, and reduced energy needs. von Haehling et al. (2010) have estimated its prevalence at 5–13% for elderly people aged 60–70 years and 11–50% for those aged 80 years or above. Lean muscle mass generally contributes up to ~50% of total body weight in young adults, but declines with aging to 25% at 75–80 years of age (Short et al., 2004). The loss of muscle mass is most notable in the lower limb muscle groups, with the cross-sectional area of the vastus lateralis being reduced by as much as 40% between the ages of 20 and 80 years (Lexell, 1995). At the muscle fiber level, sarcopenia is characterized by specific type II muscle fiber atrophy, fiber necrosis, and fiber type grouping (Lexell, 1995).

Several possible mechanisms for age-related muscle atrophy have been described.

In a recent review by Demontis et al. (2013a) provides in-depth comparison of sarcopenia in *Drosophila* and mammals. Both muscles include very similar age-related changes such as increased mitochondrial dysfunction, decreased function of autophagy/lysosome system, increased apoptosis, and protective role of dietary restriction. In contrast, aged *Drosophila* and mammalian muscles exhibit several differential characteristics (endocrine changes, decreased regenerative capacity via satellite cells, defects in Ca^2+^ homeostasis, and increased fiber atrophy). Age-related muscle loss is a result of reductions in the size and number of muscle fibers, possibly due to a multi-factorial process that involves physical activity, nutritional intake, metabolic homeostasis, oxidative stress, hormonal changes, and lifespan (Baumgartner et al., 1999; Roubenoff and Hughes, 2000; Demontis et al., 2013b). The specific contribution of each of these factors is unknown, but there is emerging evidence using rodent muscle that the distribution of several positive regulators (Akt and SRF) of muscle hypertrophy with age is an important feature in the progression of sarcopenia (Sakuma and Yamaguchi, 2010, 2011a). Very intriguingly, more recent studies indicated an apparent functional defect in autophagy- and myostatin-dependent signaling in both mice and human sarcopenic muscle (Wohlgemuth et al., 2010; McKay et al., 2012; Zhou et al., 2013). In contrast, many investigators have failed to demonstrate age-related enhancement in the levels of common negative regulators [atrophy gene-1 (atrogin-1), NF-κB, and calpain] in senescent mammalian muscles (Sakuma and Yamaguchi, 2011a, 2012c). Currently available data show that human sarcopenia is attenuated by resistance training, the ingestion of amino acids, and treatment with testosterone (Sakuma and Yamaguchi, 2011a, 2013b; Wakabayashi and Sakuma, 2014). In addition, myostatin signaling inhibition for mice and calorie restriction for mice and rhesus monkey have been shown to counteract sarcopenia (Sakuma and Yamaguchi, 2011a, 2013b). Among this, resistance training in combination with amino acid-containing nutrition is the best candidate to attenuate age-related muscle wasting and weakness in human.

### Muscular dystrophy

The neuromuscular disorders are a heterogeneous group of genetic diseases, causing progressive loss of motor ability. More than 30 genetically defined forms are recognized, and in the last decade, mutations in several genes that result in the deficiency or loss of function of various important muscle-proteins have been reported. These include dystrophin, sarcoglycans (SG), and dysferlin, which are sarcolemmal or peri-sarcolemmal proteins; α2-laminin and collagen VI, which are extracellular matrix proteins; and emerin and lamin A/C, which are nuclear proteins.

Defects in components of the dystrophin–glycoprotein complex (DGC) are known to be an important cause of different forms of muscular dystrophy (Yoshida and Ozawa, 1990; Ervasti and Campbell, 1993). The DGC is an oligomeric complex that connects the subsarcolemmal cytoskeleton to the extracellular matrix. It consists of dystroglycan (α- and β-DG), SG, and syntrophin/dystrobrevin subcomplexes. Mutations in the dystrophin gene cause the most common form of X-linked Duchenne muscular dystrophy (DMD) (Hoffman et al., 1990). The sarcoglycan sub-complex is also linked to β-DG and includes α-SG, β-SG, γ-SG, and δ-SG, which are tightly associated and inserted into the membrane. Mutations in the genes coding these four SG proteins cause severe forms of limb-girdle muscular dystrophies types LGMD2D, 2E, 2C, and 2F, respectively. α-DG, a receptor for the heterodimeric basement membrane protein laminin-2, binds to β-DG. Mutations in the LAMA2 gene, encoding the α2 chain of laminin-2, cause α 2-laminin deficiency and a severe form of congenital muscular dystrophy (CMD1A) linked to chromosome 6q (Helbling-Leclerc et al., 1995). Other milder forms of muscular dystrophy are caused by mutations in genes coding the enzyme calpain 3 (LGMD2A), the sarcolemmal protein dysferlin (LGMD2B), and the sarcomeric protein telethonin (LGMD2G) (Vainzof et al., 2008).

Sarcopenia and muscular dystrophy possess several similar characteristics as pointed out in more recent review by Rudolf et al. (2014). Fiber size variability is a major feature of various muscular dystrophy (Engel and Ozawa, 2004; Taniguchi et al., 2006; Krag et al., 2011), although it is frequently observed in sarcopenic mammalian muscles (Berger and Doherty, 2010; Hepple, 2012). The occurrence of small fiber groups was reported for samples from Becker muscular dystrophy (BMD) and DMD (ten Houten and De Visser, 1984; Engel and Ozawa, 2004), whereas elderly muscle exhibits extensive fiber type grouping (Kanda and Hashizume, 1989; Andersen, 2003). Rudolf et al. (2014) also indicated co-expression of multiple myosin heavy chain isoforms in these two muscles (Marini et al., 1991; Patterson et al., 2006). Furthermore, both muscles exhibit centralized nuclei, and the accumulation of fibrosis and intramuscular adipocyte. Although the exact reason for such a similarity has not been precisely elucidated, it seems to be feasible to apply same therapeutic approaches to sarcopenia and muscular dystrophy.

## Phosphatidylinositol-3-Kinase/Akt/Mammalian Target of Rapamycin

A central pathway involved in hypertrophy is regulated at the translational level by the serine/threonine kinase Akt. In muscle, Akt is activated by the upstream PI3-K, induced either by receptor binding or by integrin-mediated activation of focal adhesion kinase (FAK), such as in cardiac myocytes (Sakamoto et al., 2002). The striking effect of Akt1 on muscle size was demonstrated by the transient transfection of a constitutively active inducible Akt1 transgene in skeletal muscle *in vivo* (Lai et al., 2004). In addition, muscle mass was completely preserved in denervated transgenic Akt mice (Pallafacchina et al., 2002). Possible downstream regulators of Akt, mTOR, and glycogen synthase kinase (GSK)-3β, play a crucial role in the regulation of translation.

Mammalian target of rapamycin exists in two functionally distinct multi-protein signaling complexes, mTOR signaling complex (mTORC)1 and mTORC2. Akt activates mTOR via phosphorylation and inactivation of tuberous sclerosis complex (TSC)-2. In general, only signaling by mTORC1 is inhibited by rapamycin, and thus the growth regulatory effects of rapamycin are believed to be primarily exerted through the mTORC1 complex (Zoncu et al., 2011). It is now widely accepted that signaling by mTORC1 is involved in the regulation of several anabolic processes including protein synthesis and ribosome biogenesis, as well as catabolic processes such as autophagy (Zoncu et al., 2011). In skeletal muscle, signaling by mTORC1 has been shown to be regulated by a variety of different stimuli that control skeletal muscle mass. For example, signaling by mTORC1 is activated in response to hypertrophic stimuli such as increased mechanical loading (mechanical overloading for the plantaris muscle of mice by surgical ablation), feeding, and growth factors (Bodine et al., 2001b; Drummond et al., 2009).

Since signaling through PI3-K/Akt can regulate mTOR-independent growth regulatory molecules such as GSK-3β, tuberin (TSC-2), and the forkhead box O (FOXO) transcription factors (Sandri, 2008), it was not clear whether signaling by mTORC1 is sufficient, or simply permissive, for the induction of hypertrophy. For example, Hornberger et al. (2003) found that stretch-induced activation of mTOR signaling was not abolished in the skeletal muscle of Akt1−/− mice. Furthermore, Akt-independent stimulation of mTOR may be positively or negatively regulated by phosphorylation of TSC-2. For instance, TSC-2 is inhibited by FAK in 293T cells (Gan et al., 2006), suggesting that up-regulation of FAK expression with increased mechanical loading for skeletal muscle could stimulate protein synthesis via TSC-2 inhibition. All these regulatory influences may explain the rise in the level of phosphorylated p70S6K (Coffey et al., 2006). Therefore, mTOR is currently thought to be the major hub for the integration of an array of upstream signaling pathways that, when activated, ultimately result in increased translational efficiency (Glass, 2010).

Two of the most studied mTORC1 targets are the eukaryotic initiation factor 4E binding protein (4E-BP)1 and p70S6K, which both play important roles in the initiation of mRNA translation. mTOR phosphorylates and activates the 70-kDa ribosomal protein S6 kinase (p70S6K), which results in increased translation either directly or indirectly by activating initiation and elongation, elongation initiation factor (eIF)-2, eIF4E (through 4E-BP), and eEF-2 (Glass, 2010). In addition, Akt also phosphorylates and inactivates GSK-3β, thereby activating translation via the initiation factor eIF2B. Other functions of Akt include the negative regulation of protein degradation by inhibiting FOXO-mediated proteasome activity.

Demontis and Perrimon (2009) showed that insulin receptor signaling and FOXO can regulate skeletal muscle atrophy also in *Drosophila* larval muscle. This study shows evolutionarily conservation of the mechanisms controlling muscle atrophy. It also shows a role for the transcription factors Myc and Mnt in this process (these are new factors that were not known to be involved in this process in mice or humans). Therefore, it is probable for the existence of novel signaling pathway via FOXO to regulate muscle hypertrophy and/or atrophy in mammals.

### Adaptation of PI3-K/Akt/mTOR pathway in aged muscle

Although many researchers consider PI3-K/Akt/mTOR levels to decrease with age, studies using sarcopenic muscles from rats and humans have yielded conflicting results. For example, compared with those in young Fischer 344 × Brown Norway rats, the amounts of phosphorylated mTOR and p70S6K were increased 70–75% in the tibialis anterior (TA) but not in the plantaris muscle of senescent rats (Parkington et al., 2004). Kimball et al. (2004) showed that, in gastrocnemius muscle, the level of phosphorylated p70S6K, eIF2B activity, and the amount of eIF4E associated with eIF4G increased between 12 and 27 months of age despite an apparent decrease in Akt activity. In addition, other groups (Haddad and Adams, 2006; Léger et al., 2008) also showed the decreased phosphorylation status of Akt in aged mammalian muscle. In contrast, Rahnert et al. (2011) showed only significant decrease of phospho-p70S6K (T^421^/S^424^) in the aged biceps brachii and no change in phospho-p70S6K (T^389^), in spite of significant age-related decrease in p70S6K in all head and neck, tongue, and limb muscles (pectoralis, styloglossus, geniohyoid, posterior digastric, and masseter). Therefore, aging did not commonly modulate the PI3-K/Akt/mTOR-linked molecules in skeletal muscle under sedentary conditions.

Sarcopenic muscle shows a marked defect in the contraction-induced activation of these mediators. Parkington et al. (2004) reported lower levels of phosphorylated p70S6K and mTOR after high-frequency electrical stimulation [HFES, 3-s trains of pulses (frequency 100 Hz, duration 1 ms at 10–12 V)] in muscle of senescent rats (30 months of age) compared with those in young rats (6 months of age). The same roup (Funai et al., 2006) also demonstrated that 4E-BP1 was markedly phosphorylated in the TA muscle of aged but not young rats at 6 h after HFES. In addition, they suggested no increase in eIF4E–eIF4G association after HFES in aged muscle (Funai et al., 2006). Furthermore, Thomson and Gordon (2006) suggested impaired overload-induced muscle growth in old rats possibly due to diminished phosphorylation of mTOR (Ser^2448^), p70S6K (mTOR-specific Thr^389^), rpS6 (Ser^235/236^), and 4E-BP1. Fry et al. (2011) demonstrated that acute resistance exercise (8 sets of 10 repetitions of leg-extension at 70% 1RM with 3 min of rest between each set) increased muscle-protein synthesis rate, and phosphorylation of mTOR, S6K1, and 4E-BP1 only in younger subjects (27 ± 2 years old) but not in elderly ones (70 ± 2 years old). These lines of evidence clearly show that sarcopenic muscle exhibits an impairment of Akt/mTOR/p70S6K signaling after contraction. This defect would explain the limited capacity for hypertrophy after muscle stimulation in aged animals.

### Adaptation of the PI3-K/Akt/mTOR pathway in dystrophic muscle

Functional deficiency of mTOR-dependent signaling is implicated in muscular dystrophy. Indeed, muscles lacking raptor (mTORC1 component) but not rictor (mTORC2 component) become progressively dystrophic and kyphotic, resulting in early death (Bentzinger et al., 2008). In the soleus and to a lesser extent in the EDL, raptor-deficient mice exhibited a wide distribution of fiber size, muscle fibers with centralized nuclei, and structures reminiscent of central cores (Bentzinger et al., 2008). Dystrophic muscle seems to exhibit induction of this anabolic pathway. Compared with age-matched wild-type mice, marked increases in pAkt/Akt, pS6/S6, and p4E-BP1/4E-BP1 were recognized in TA and diaphragm muscles of 4-month-old mdx mice (De Palma et al., 2012). Intriguingly, starvation was shown to elicit significant decreases in these anabolic mediators of mTOR-dependent signaling in both muscles of wild-type mice, but not those of mdx mice. Such hyperactivation of this signal markedly blocks autophagy-dependent signaling in both normal and starved mdx mice (De Palma et al., 2012). Age-related reductions of pAkt and pS6 levels occur in mdx mouse muscle. Indeed, Mouisel et al. (2010) showed marked decreases in pAkt (50%) and pS6 (45%) in mdx muscle at 18–24 months old compared with those at 5 months old. Intriguingly, the stimulation of muscle regeneration by cardiotoxin injury induces abnormal hyperactivation of pAkt and pS6. Therefore, sarcopenia muscle of mdx mice exhibits an apparent deficiency of PI3-K/Akt/mTOR signaling. However, as mdx mice age normally, caution is required when translating observations from mdx mice to human DMD patients. In addition, they similarly observed hyperactivation of pAkt and p4E-BP1, no induction of LC3-II, and accumulation of p62 in muscles of DMD patients. At 6 weeks of age, there was a significantly lower level of mTOR activation in diaphragm muscles of mdx mice compared with that of age-matched wild-type mice (Eghtesad et al., 2011). mTOR activation increased with postnatal age in diaphragm muscle of wild-type mice, but not in mdx mice. In contrast to diaphragm muscle, mTOR activation was not significantly different in the TA muscle of mdx and wild-type mice at either 6 or 12 weeks of age (Eghtesad et al., 2011). As contradicting results relating to the adaptive changes in PI3-K/Akt/mTOR in muscular dystrophy have been observed, future studies using human patients with muscular dystrophy are required. Strangely, a low-protein diet (De Palma et al., 2012) and treatment with rapamycin (Eghtesad et al., 2011) attenuate this anabolic pathway, but Wnt7a (von Maltzahn et al., 2012) and valproic acid (Gurpur et al., 2009) activate it. However, such therapeutics with overall different directions for mTOR-dependent signaling effectively attenuates the muscular dystrophic phenotype (muscle inflammation such as T-cell infiltration, fibrosis, myofiber damage, and the decrease of muscle strength).

## Serum Response Factor

Serum response factor is a ubiquitously expressed member of the MADS (MCM1, Agamous, Deficiens, and SRF) box transcription factor family, sharing a highly conserved DNA-binding/dimerization domain, which binds the core sequence of SRF/CArG boxes [CC (A/T)6 GG] as homodimers. SRF-dependent signaling plays a major role in a variety of physiological processes, including cell growth, migration, and cytoskeletal organization (Pipes et al., 2006). Previous results obtained with specific SRF-knockout models by the Cre–LoxP system emphasize a crucial role for SRF in postnatal skeletal muscle growth and regeneration by modulating interleukin-4 and IGF-I (insulin-like growth factor-I) mRNA expression (Charvet et al., 2006). More recently, Mokalled et al. (2012) demonstrated that members of the myocardin family of transcriptional coactivators, MASTR, and myocardin-related transcription factor (MRTF)-A, are up-regulated in satellite cells in response to skeletal muscle injury. In addition, double-knockout satellite cells (MASTR and MRTF-A) impair skeletal muscle regeneration, probably due to the down-regulation of several modulators of cell cycle arrest (retinoblastoma, etc.). As proposed by Mokalled et al. (2012), the promoting role on muscle regeneration seems to be attributable to both MASTR/MEF2 and/or MRTF–A/SRF complexes because the mouse MASTR protein lacks SRF-interaction regions.

Serum response factor also enhances the hypertrophic process in muscle fibers after mechanical overloading (Gordon et al., 2001; Sakuma et al., 2003; Sakuma and Yamaguchi, 2012a, 2013a) as well as muscle differentiation and MyoD gene expression *in vitro* (Gauthier-Rouviére et al., 1996). Although SRF would regulate proliferation and differentiation using different pathways, it would mainly activate the differentiation of satellite cells during muscle hypertrophy. Indeed, we showed that, in mechanically overloaded muscles of rats, the SRF protein co-localized with MyoD and myogenin in myoblast-like cells during the active differentiation phase (Sakuma et al., 2003). More recently, Guerci et al. (2012) investigated the functional role of SRF in fiber hypertrophy using SRF^flox/flox^:HAS-Cre-ER^T2^ mice injected with tamoxifen. Guerci et al. (2012) showed that the selective lack of SRF in myofibers markedly slows fiber growth after mechanical overloading by modulating satellite cell proliferation and fusion to the growing fibers. They demonstrated that, in the overloaded muscle, SRF enhances the expression of COX2 mRNA, which in turn upregulates IL-4 mRNA and ultimately secretes IL-4 protein. Guerci’s hypothesis indicated that IL-4 produced by muscle fibers moves into satellite cells paracrinally to modulate the fusion of satellite cells.

It is proposed that the transcriptional activity of SRF is regulated by muscle ring finger (MuRF)-2 (Lange et al., 2005) and striated muscle activators of Rho signaling (STARS) (Kuwahara et al., 2005). At the M-band, the mechanically modulated kinase domain of titin interacts with a complex of the protein products of the atrogenes NBR1, p62/SQSTM-1, and MuRFs (Lange et al., 2005; Puchner et al., 2008). This complex dissociates under mechanical arrest, and MuRF-1 and MuRF-2 translocate to the cytoplasm and the nucleus (Lange et al., 2005; Ochala et al., 2011). One of the probable nuclear targets of MuRFs is SRF (Lange et al., 2005), suggesting that the MuRF-induced nuclear export and transcriptional repression of SRF may contribute to amplifying the transcriptional atrophy program (Spencer et al., 2000). Thus, it is possible that the synergistic transactivation of SRF and SRF-linked molecules is abrogated by MuRF-2 *in vivo*. On the other hand, SRF activity is exquisitely sensitive to the state of actin polymerization. G-actin monomers inhibit SRF activity, whereas polymerization of actin occurs in response to serum stimulation and RhoA signaling. In this pathway, signal inputs lower the ratio of globular actin to fibrillar actin, thereby liberating the binding of MRTF-A to globular actin, resulting in the nuclear accumulation of MRTF-A and subsequent SRF-dependent gene expression (Miralles et al., 2003). It has been well established that overexpression of STARS contributes to the nuclear translocation of MRTF-A and MRTF-B (Kuwahara et al., 2005, 2007), and these factors activate SRF transcription.

### Adaptive changes in SRF-linked molecules with age

Mechanical loading for skeletal muscle is widely accepted to determine SRF expression. In humans, Lamon et al. (2009) demonstrated that 8 weeks of resistance training (leg presses, squats, and leg-extensions) induced increases in SRF mRNA (3-fold) and nuclear protein (1.25-fold) in the vastus lateralis muscle. In the same training period, they also observed a similar increase in the mRNA levels of several SRF-targeted molecules (alpha-actin, myosin heavy chain IIa, and IGF-I) (Charvet et al., 2006). Using RT-PCR, crude and fractionated homogenates, and immunofluorescence, our study demonstrated blunted expression of SRF protein in the quadriceps and triceps brachii muscles in aged mice (Sakuma et al., 2008). Immunofluorescence microscopy also indicated the selective down-regulation of SRF immunoreactivity in the cell cytosol but not in Pax7-labeled satellite cells in sarcopenic mice. In addition, our data showed a decrease in MRTF-A mRNA (50–70%) and protein (76%) levels in only the nuclear fraction with age. Furthermore, 60 and 40% decreases in the amount of STARS mRNA were observed in the quadriceps and triceps brachii of 24-month-old mice, respectively (Sakuma et al., 2008). Intriguingly, a decrease of SRF expression achieved by a transgenic approach using the Cre–LoxP system was found to accelerate the atrophic process in muscle fibers with age (Lahoute et al., 2008). These SRF KO mice showed marked deposition of intramuscular lipids with aging. One morphologic aspect of sarcopenia is the infiltration of muscle tissue components by lipids because of the increased frequency of adipocyte or lipid deposition (Dubé and Goodpaster, 2006) within muscle fibers. As with precursor cells in bone marrow, liver, and kidney, muscle satellite cells expressing the adipocytic phenotype increased with age (Shefer et al., 2006), although this process is still relatively poorly understood in terms of its extent and spatial distribution. Lipid deposition, often referred to as intramuscular lipid deposition, may result from a net buildup of lipids due to the reduced oxidative capacity of muscle fibers with aging (Dubé and Goodpaster, 2006). These lines of evidence clearly show the existence of a defect of SRF signaling in aged mammalian muscle.

### Adaptive changes in SRF-linked molecules with muscular dystrophy

Serum response factor appears to be linked to the degenerative process during muscular dystrophy. Significant reductions in the amount of SRF have been observed (Sakuma et al., 2004), namely, 40–50 and 50–65% at 2 and 12 weeks of age, respectively, in merosin-deficient congenital muscular dystrophy. Our immunohistochemical analysis indicated that mature normal mice had an abundance of SRF protein in the cytoplasm of several muscle fibers, while the dy mice did not. In the skeletal muscle, there is no direct evidence of a link between SRF disorders and the pathogenesis of disease. However, Lange et al. (2005) observed that a mutation in the TK domain of titin, a possible upstream modulator of SRF, disrupted Nbr1 binding, and led to hereditary myopathy with early respiratory failure (HMERF). HMERF patient biopsies revealed diffusible localization of Nbr1, large cytoplasmic aggregates of p62, and the selective accumulation of MuRF-2 in centralized nuclei in diseased muscle. Unfortunately, their study did not examine the localization of SRF in the muscle of HMERF patients. In contrast, human heart failure was reported to show elevations of a natural dominant-negative form of SRF arising from alternative splicing (Davis et al., 2002). The dominant-negative SRF isoform potently inhibited SRF-dependent gene expression, mirroring the biochemical phenotype seen in SRF-null mice (Davis et al., 2002). In addition, a subsequent human heart failure study showed decreases in full-length SRF and elevated expression of a caspase-3-cleaved product of SRF (Chang et al., 2003). A more recent review (Miano, 2010) proposed various disorders to be linked with the SRF mutations as shown by many reliable studies using cell-specific SRF-knockout phenotypes.

## Ubiquitin–Proteasome System

The ATP-dependent UPS is essential for regulating protein degradation. The degradation of a protein via the UPS involves two steps: (1) tagging of the substrate by covalent attachment of multiple ubiquitin molecules and (2) degradation of the tagged protein by the 26S proteasome complex with the release of a free and reusable ubiquitin. Ubiquitin, composed of 76 amino acids, is an 8.45-kDa protein that is highly conserved in nearly all eukaryotes. The ubiquitination of proteins is regulated by at least three enzymes: ubiquitin-activating enzyme (E1); ubiquitin-conjugating enzyme (E2); and ubiquitin ligase (E3). Kwak et al. (2004) suggested that the 14-kDa ubiquitin-conjugating enzyme E2_14K_ and the ubiquitin ligase E3 are particularly important for the degradation of muscle-proteins. The labeled proteins are then fed into the cells’ “waste disposers,” the proteasomes, where they are chopped into small pieces and destroyed.

Atrogin-1 is a member of the Skp1, Cullin 1, and F-box-containing protein (SCF) complex, which bind together to establish E3 Ub-protein ligase activity, and features an approximately 40-amino-acid motif known as an F-box. MuRF-1 contains a canonical N-terminal RING domain characteristic of RING-containing E3 ligases followed by a MuRF family conserved region, zinc-finger domain (B-box), and leucine-rich coiled-coil domains. Consistent increases in atrogin-1 and MuRF-1 gene expression have been observed in a wide range of *in vivo* models of skeletal muscle atrophy including diabetes, cancer, renal failure, denervation, unweighting, and glucocorticoid or cytokine treatment (Bodine et al., 2001a; Lecker et al., 2004). The importance of these atrophy-regulated genes in muscle wasting was confirmed through knockout studies in mice where an absence of atrogin-1 or MuRF-1 attenuated denervation-, fasting-, and dexamethasone-induced muscle atrophy (Bodine et al., 2001a; Baehr et al., 2011; Cong et al., 2011).

Yeast two-hybrid analysis identified eIF3 subunit 5 (eIF3-f) and MyoD as interactors of atrogin-1 (Lagirand-Cantaloube et al., 2008, 2009). Conversely, the knockdown of atrogin-1 reversed endogenous MyoD proteolysis and the overexpression of a mutant MyoD, unable to be ubiquitinated, prevented muscle atrophy *in vivo* (Lagirand-Cantaloube et al., 2009). These results confirmed MyoD as a substrate of atrogin-1, resulting in its polyubiquitination and subsequent degradation during dexamethasone-induced myotube atrophy (Jogo et al., 2009). In the heart, atrogin-1 ubiquitinates and reduces the levels of calcineurin A, an important factor triggering cardiac hypertrophy in response to pressure overload (Li et al., 2004). Interestingly, immunoprecipitation experiments in C2C12 myoblasts and myotubes have found that atrogin-1 interacts with sarcomeric proteins, including myosins, desmin, and vimentin, as well as transcription factors, components of the translational machinery, enzymes involved in glycolysis and gluconeogenesis, and mitochondrial proteins (Lokireddy et al., 2012). Whether atrogin-1 ubiquitinates these proteins has yet to be proven. In contrast to atrogin-1, it appears that MuRF-1 mainly interacts with structural proteins. MuRF-1 was reported to interact with and control the half-life of many important muscle structural proteins, including troponin I, titin, myosin heavy chain (Clarke et al., 2007), actin (Polge et al., 2011), myosin binding protein C, and myosin light chains 1 and 2 (Cohen et al., 2009). For example, MuRF-1 degrades myosin light chains 1 and 2 under denervation and fasting conditions (Cohen et al., 2009). These studies suggest that, while numerous stimuli can activate both atrogin-1 and MuRF-1, the downstream pathways affected may be separate for each protein.

### Adaptation of UPS in aged muscle

Only very indirect measurements [small increases in levels of mRNA encoding some components of the UPS (Bossola et al., 2008; Combaret et al., 2009) or ubiquitin-conjugate accumulation] in old muscles of rodents or humans suggested modest activation of this pathway. Atrogin-1 and/or MuRF-1 mRNA levels in aged muscle are reportedly increased (Clavel et al., 2006) or unchanged (Welle et al., 2003; Whitman et al., 2005) in humans and rats, or decreased in rats (DeRuisseau et al., 2005; Edström et al., 2006). Even when the mRNA expression of these atrogenes increased in sarcopenic muscles, this was very limited (1.5- to 2.5-fold) compared with that in other catabolic conditions (10-fold).

Although various findings have been made regarding the mRNA levels of both ubiquitin ligases in aged mammalian muscle, the examination of protein levels in sarcopenic muscles did not support age-related increases in the mRNA of several ubiquitin ligases. For instance, Edström et al. (2006) indicated the marked up-regulation of phosphorylated Akt and FOXO4 in the gastrocnemius muscle of aged female rats, probably contributing to the down-regulation of atrogin-1 and MuRF-1 mRNA. This result is further supported by the more recent finding of Léger et al. (2008) who, using human subjects aged 70 years old, demonstrated decreases in nuclear FOXO1 and FOXO3a by 73 and 50%, respectively, although they did not recognize significant age-dependent changes in the expression of atrogin-1 and MuRF-1 mRNA. The major peptidase activities of the proteasome (i.e., the chymotrypsin-like, trypsin-like, and caspase-like activities) were either reduced (as reported in other tissues) or unchanged with aging (Combaret et al., 2009; Sakuma and Yamaguchi, 2011a). In contrast, Altun et al. (2010) recently found that the hindlimb muscles of (30-month-old) rats contained two to threefold more 26S proteasomes than purified from muscles of aged rats, and adult (control) rats showed a similar capacity to degrade peptides, proteins, and a ubiquitinated substrate, but differed in the levels of proteasome-associated proteins (e.g., the deubiquitinating enzyme USP14). Although the activities of many other deubiquitinating enzymes were greatly enhanced in aged muscles, levels of polyubiquitinated proteins were higher than in the adult animals. Interestingly, recent findings indicate that atrogin-1-knockout mice are short-lived and experience higher loss of muscle mass during aging than control mice (Sandri et al., 2013), indicating that the activity of this E3 ubiquitin ligase is required to preserve muscle mass during aging in mice. Moreover, MuRF-1-null mice experience higher decay of muscle strength during aging than controls, although muscle mass is at least in part preserved in these mice (Hwee et al., 2014). As indicated by Sandri et al. (2013), chronic inhibition of these atrogenes should not be considered a therapeutic target to counteract sarcopenia because this does not prevent muscle loss but instead exacerbates weakness.

### Adaptation of UPS in muscular dystrophy

Gene expression profiling in LGMD2A showed overexpression of UPS-related genes (Keira et al., 2007; Saenz et al., 2008). While the expression of atrogin-1 and MuRF-1 was not increased in mouse models of LGMD2A, FOXO1 was strongly up-regulated, and induced muscle atrophy in calpain-3-deficient mice (Laure et al., 2009). More recently, Fanin et al. (2013) demonstrated that LGMD2A patients exhibit significantly higher expression of MuRF-1 protein (146 ± 64% of control) but not atrogin-1 protein (77 ± 26% of control) in skeletal muscle.

LGMD2B is due to deficiency of the protein dysferlin, which causes failure in resealing of the membrane lesions generated during eccentric muscle contractions (Bansal et al., 2003). Similar to LGMD2A, dysferlinopathy patients exhibited more abundant mRNA and protein of MuRF-1 but not atrogin-1 (Fanin et al., 2014). Activation of UPS in dysferlinopathy has also been reported in cellular models (patient-derived muscle cells) (Azakir et al., 2012). Ullrich congenital muscular dystrophy (UCMD) is a common form of muscular dystrophy associated with defects in collagen VI. It is characterized by loss of individual muscle fibers and muscle mass and proliferation of connective and adipose tissues. More recently, Paco et al. (2012) studied muscle biopsies of UCMD (*n* = 6), other myopathy (DMD, calpain-3-deficient, Kearns–Sayre, and nemaline myopathy, *n* = 12), and control patients (*n* = 10) and found reduced expression of atrogin-1 and MuRF-1 mRNAs in UCMD cases.

In contrast to the case of sarcopenia, pharmacological inhibition of UPS appears to exert some beneficial effect on muscular dystrophy. Bonuccelli et al. (2007) indicated that Velcade, once injected locally into the gastrocnemius muscles of mdx mice, could upregulate the expression and membrane localization of dystrophin and members of the DAPC. Gazzerro et al. (2010) suggested that treatment with Velcade (0.8 mg/Kg) over a 2-week period reduced muscle degeneration and necrotic features, and increased muscle size (gastrocnemius and diaphragm), in mdx muscle fibers. In addition, they observed many myotubes and/or immature myofibers expressing embryonic myosin heavy chain in mdx muscle after Velcade administration, probably due to up-regulation of several myogenic differentiating modulators (MyoD and Myf-5). They also demonstrated that MG-132 increased dystrophin, α-sarcoglycan, and β-dystroglycan levels in explants from BMD patients, whereas it increased levels of the DAPC in DMD cases.

## Autophagy-Dependent Signaling

Macroautophagy (herein autophagy) occurs in all eukaryotic cells and is evolutionarily conserved from yeast to humans. Autophagy is a ubiquitous catabolic process that involves the bulk degradation of cytoplasmic components through a lysosomal pathway (Sandri, 2010, 2011; Neel et al., 2013). This process is characterized by the engulfment of part of the cytoplasm inside double-membrane vesicles called autophagosomes. Autophagosomes subsequently fuse with lysosomes to form autophagolysosomes in which the cytoplasmic cargo is degraded and the degradation products are recycled for the synthesis of new molecules. Turnover of most long-lived proteins, macromolecules, biological membranes, and whole organelles, including mitochondria, ribosomes, the endoplasmic reticulum, and peroxisomes, is mediated by autophagy (Cuervo, 2004).

At first glance, autophagy was considered a coarse, non-selective, degradative system, but closer investigation revealed a different truth. Autophagy represents an extremely refined collector of altered organelles, abnormal protein aggregates, and pathogens, similar to a selective recycling center rather than a general landfill (Park and Cuervo, 2013). The selectivity of the autophagy process is conferred by a growing number of specific cargo receptors, including p62/SQSTM-1, Nbr1, Nix (Bnip3L), and optineurin (Shaid et al., 2013). These adaptor proteins are equipped with both a cargo-binding domain, with the capability to recognize and attach directly to molecular tags on organelles, and at the same time an LC3-interacting region domain, able to recruit and bind essential autophagosome membrane proteins.

*De novo* formation of autophagosomes is regulated by at least three molecular complexes: the LC3 conjugation system and the regulatory complexes governed by unc51-like kinase-1 (ULK1) and Beclin-1. The conjugation complex is composed of different proteins encoded by autophagy-related genes (Atg) (Mizushima and Komatsu, 2011). The Atg12–Atg5–Atg16L1 complex, along with Atg7, plays an essential role in the conjugation of LC3 to phosphatidylethanolamine, which is required for the elongation and closure of the isolation membrane (Mizushima and Komatsu, 2011). This system is under the regulation of at least two major cellular energy-sensing complexes. Under basal conditions, the ULK1 complex is inactivated by phosphorylation through mTORC1, whereas during autophagy induction mTORC1 is inhibited, thus enhancing the formation of a complex between ULK1, Atg13, and FIP200. In addition, mTORC1 can also be negatively regulated independently of Akt by energy stress sensors such as AMPK and, in a mechanical-activity-dependent manner, through TSC-1/2. Moreover, AMPK can also directly phosphorylate ULK1 and Beclin-1 (Kim et al., 2013). During autophagy, the ULK1 complex is localized to the isolation membrane, where it facilitates the formation of autophagosomes through interaction with the Beclin-1 complex.

Interestingly, that the UPS and the lysosomal–autophagy system in skeletal muscle are interconnected was suggested by Mammucari et al. (2007), and Zhao et al. (2007). Both studies identified FOXO3 as a regulator of the lysosomal and proteasomal pathways in muscle wasting. FOXO3 is a transcriptional regulator of the ubiquitin ligases MuRF-1 and atrogin-1. It has now been linked to the expression of Atg in skeletal muscle *in vivo* and C2C12 myotubes (Zhao et al., 2007). More recently, Masiero et al. (2009) found an intriguing characteristic using muscle-specific autophagy-related gene (Atg7) knockout mice. The atrophy, weakness, and mitochondrial abnormalities in these mice are also features of sarcopenia.

### Adaptation of autophagy-linked signaling in muscle with age

A decline in autophagy during normal aging has been described for invertebrates and higher organisms (Cuervo et al., 2005). Inefficient autophagy has been attributed a major role in the apparent age-related accumulation of damaged mitochondria (Terman and Brunk, 2006).

Demontis and Perrimon (2010) showed that the function of autophagy/lysosome system of protein degradation declined during aging in the skeletal muscle of *Drosophila*. This results in the progressive accumulation of polyubiquitin protein aggregates in senescent *Drosophila* muscle. Intriguingly, overexpression of the FOXO increases the expression of many autophagy genes, preserves the function of the autophagy pathway, and prevents the accumulation of polyubiquitin protein aggregates in sarcopenic *Drosophila* muscle (Demontis and Perrimon, 2009). Several investigators reported the autophagic changes in aged mammalian skeletal muscle (McMullen et al., 2009; Wenz et al., 2009; Wohlgemuth et al., 2010; Gaugler et al., 2011). Compared with those in young male Fischer 344 rats, amounts of Beclin-1 were significantly increased in the plantaris muscles of senescent rats (Wohlgemuth et al., 2010). In contrast, aging did not influence the amounts of Atg7 and Atg9 proteins in rat plantaris muscle (Wohlgemuth et al., 2010). Indeed, Western blot analysis by Wohlgemuth et al. (2010) clearly showed a marked increase in the amount of LC3 in muscle during aging. However, they could not demonstrate an aging-related increase of the ratio of LC3-II to LC3-I, a better biochemical marker to assess ongoing autophagy. In contrast, Wenz et al. (2009) recognized a significant increase in the ratio of LC3-II to LC3-I during aging (3 vs. 22 months) in the biceps femoris muscle of wild-type mice. None of the studies determining the transcript level of autophagy-linked molecules found a significant increase with age (McMullen et al., 2009; Wohlgemuth et al., 2010; Gaugler et al., 2011). Not all contributors to autophagy signaling seem to change similarly at both mRNA and protein levels in senescent skeletal muscle. Therefore, sarcopenia may include a partial defect of autophagy signaling, although more exhaustive investigation is needed in this field.

Life-long caloric restriction alone, or combined with voluntary exercise, resulted in mild reduction of LC3 expression and lipidation coupled with increased LAMP-2 (lysosomal marker) expression, suggesting a potential increase in autophagy flux. No significant age-related increase in autophagy-linked molecules was observed in MCK-PGC-1α mice. PGC-1α may also enhance autophagic flux. More recently, GSK-3α was proposed as a critical regulator of aging in various organs (skeletal muscle, heart, liver, bone, etc.) via modulating mTORC1 and autophagy. Intriguingly, mice with null mutation of GSK-3α showed premature death and acceleration of age-related pathologies such as vacuolar degeneration, large tubular aggregates, sarcomere disruption, and striking sarcopenia in cardiac and skeletal muscle (Zhou et al., 2013). These GSK-3α KO mice exhibited marked activation of mTORC1 and associated suppression of several autophagy molecules. Indeed, unrestrained activation of mTORC1 leads to profound inhibition of autophagy (Levine and Kroemer, 2008; Kroemer et al., 2010). Therefore, it is expected that pharmacological inhibition (everolimus) of mTORC1 rescued the muscular disorder resembling sarcopenia in GSK-3α KO mice (Zhou et al., 2013). Enhancement of autophagy flux (exercise, caloric restriction, etc.) would be a potential strategy attenuating sarcopenia as well as various type of muscular dystrophy with autophagy defect (Grumati et al., 2010; De Palma et al., 2012; Vainshtein et al., 2014).

### Adaptation of autophagy-linked signaling in muscular dystrophy

A finely tuned system for protein degradation and organelle removal is required for the proper function and contractility of skeletal muscle (Vainshtein et al., 2014). Inhibition/alteration of autophagy contributes to myofiber degeneration leading to accumulation of abnormal (dysfunctional) organelles and of unfolded and aggregation-prone proteins (Masiero et al., 2009; Sandri, 2010), which are typical features of several myopathies (Grumati et al., 2010; Nogalska et al., 2010). Generation of Atg5 and Atg7 muscle-specific knockout mice confirmed the physiological importance of the autophagy system in muscle mass maintenance (Raben et al., 2008; Masiero et al., 2009). The muscle-specific Atg7 knockout mice are characterized by the presence of abnormal mitochondria, oxidative stress, accumulation of polyubiquitinated proteins, and consequent sarcomere disorganization (Masiero et al., 2009). In addition, the central role of the autophagy–lysosome system in muscle homeostasis is highlighted by lysosomal storage diseases (Pompe disease, Danon disease, and X-linked myopathy), a group of debilitating muscle disorders characterized by alterations in lysosomal proteins and autophagosome buildup (Vainshtein et al., 2014). Intriguingly, all of these myopathies exhibit the accumulation of autophagic vacuoles inside myofibers due to defects in their clearance.

**Figure 1:**
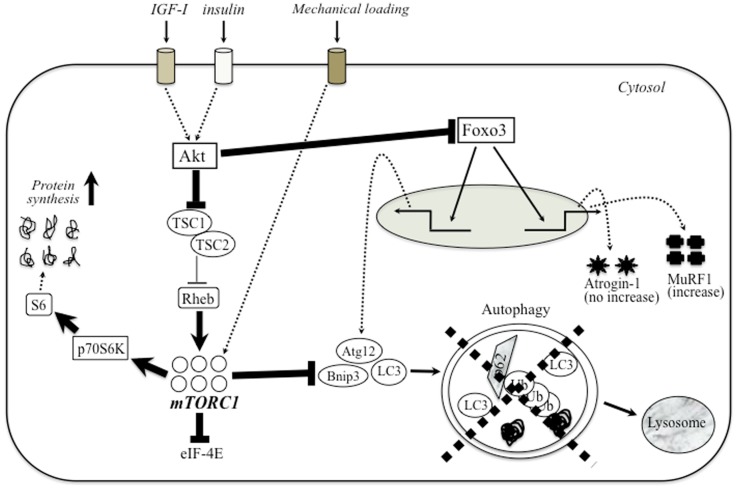
**The relationship between PI3-K–Akt–mTOR signaling and autophagy in muscular dystrophy**. The major anabolic pathway regulating protein synthesis in skeletal muscle is mTOR/TORC1 signaling. Upstream trigger (IGF-1, mechanical stress, etc.) activates mTOR signaling through a number of different intermediary proteins such as Akt and Rheb. Several anabolic stimulation increases the amount of activated Akt, which blocks the nuclear translocation of Foxo3 to enhance the expression of autophagy-related genes (Bnip, LC3, and Atg12) and atrogene (atrogin-1 and MuRF-1). In dystrophic muscle, higher Akt potently blocks the inhibition of Rheb by TSC-1/TSC-2, and hyperactivate mTORC1. Unnecessary activated mTORC1 would extremely enhance protein synthesis and blocks autophagy-dependent signaling. Therefore, muscular dystrophy exhibits apparent defect of autophagic process similar to sarcopenic muscle.

Apparent defect of autophagy-dependent signaling is also observed in various muscular dystrophies. The first evidence of impaired autophagy in these models was provided by studies in mice and patients with mutations in collagen VI (Irwin et al., 2003). Mutations that inactivate Jumpy, a phosphatase that counteracts the activation of VPS34 for autophagosome formation and reduces autophagy, are associated with centronuclear myopathy (Vergne et al., 2009). De Palma et al. (2012) have described marked defect of autophagy in dystrophin-deficient mdx mice and DMD patients. This evidence included the electron microscopic evaluation of muscle tissue morphology as well as the decreased expression of autophagic regulator proteins (i.e., LC3-II, Atg12, Gabarapl1, and Bnip3). In addition, starvation and treatment with chloroquine, potent inducers of autophagy, did not activate autophagy-dependent signaling in both TA and diaphragm muscles of mdx mice (De Palma et al., 2012). Furthermore, mdx mice and DMD patients exhibited an unnecessary accumulation of p62 protein, which was lost after prolonged autophagy induction by a low-protein diet (De Palma et al., 2012). A similar block in autophagy progression was described in lamin A/C null mice (Ramos et al., 2012). LGMD2A muscles showed up-regulation of p62 (2.1-fold) and Bnip3 (3-fold) mRNA and slightly increased LC3-II/LC3-I protein ratio and p62 (Fanin et al., 2013). Conversely, laminin-mutated (dy/dy) animals displayed excessive levels of autophagy, which is equally detrimental (Carmignac et al., 2011). These findings suggest that the defect of autophagy signaling has a central role in the degenerative symptoms in various types of muscular dystrophy. Figure 1 shows a schematic diagram of possible relationship between Akt–mTOR signaling and autophagy in muscular dystrophy.

## Myostatin

Growth and differentiation factor 8, otherwise known as myostatin, was first discovered during screening for novel members of the transforming growth factor-β (TGF-β) superfamily, and shown to be a potent negative regulator of muscle growth (Lee, 2004). Like other TGF-β family members, myostatin is synthesized as a precursor protein that is cleaved by furin proteases to generate the active C-terminal dimer. When produced in Chinese hamster ovary cells, the C-terminal dimer remains bound to the N-terminal propeptide, which remains in a latent, inactive state (Wolfman et al., 2003). Most, if not all, of the myostatin protein that circulates in the blood also appears to exist in an inactive complex with a variety of proteins, including the propeptide. Myostatin binds to and signals through a combination of ActRIIA/B receptors on the cell membrane, but has higher affinity for activin type IIB receptor (ActRIIB). On binding to ActRIIB, myostatin forms a complex with a second surface type I receptor, either activin receptor-like kinase 4 or 5, to stimulate the phosphorylation of Receptor Smad (R-Smad) and the Smad2/3 transcription factors in the cytoplasm. This leads to the assembly of Smad2/3 with Smad4 to form a heterodimer that can translocate to the nucleus and activate the transcription of target genes (Joulia-Ekaza and Cabello, 2007). Myostatin circulates in the blood in a latent complex with non-covalently bound propeptide at the N-terminus (Wolfman et al., 2003).

Studies indicate that myostatin inhibits cell cycle progression and reduces the levels of myogenic regulatory factors, thereby controlling myoblastic proliferation and differentiation during developmental myogenesis (Yang et al., 2007). One of the known downstream targets of Smad signaling is MyoD. Interestingly, myostatin downregulates MyoD expression in an NF-κB-independent way (McFarlane et al., 2006). Myostatin also inhibits Pax3 expression, which is possibly an upstream target of MyoD (McFarlane et al., 2006). On the other hand, the genetic loss of myostatin leads to an increase in Akt activity in skeletal muscle *in vivo* and *in vitro* (Morissette et al., 2009). The IGF-1–Akt–mTOR pathway, which mediates both differentiation in myoblasts and hypertrophy in myotubes, has been shown to inhibit myostatin-dependent signaling. Blockade of the Akt–mTOR pathway using siRNA to RAPTOR, a component of TOR signaling complex 1 (TORC1), facilitates myostatin’s inhibition of muscle differentiation because of an increase in Smad2 phosphorylation (Trendelenburg et al., 2009). Taking these findings, myostatin-mediated signaling activates FOXO, which leads to the expression of ubiquitin ligases.

### Adaptive changes in myostatin in sarcopenic muscle

Myostatin levels increase with muscle atrophy due to unloading in mice and humans (Wehling et al., 2000; Sakuma et al., 2009), and with severe muscle wasting in patients with cancer cachexia, chronic heart failure, chronic obstructive pulmonary disease (COPD), AIDS, and diabetes (Sakuma and Yamaguchi, 2011b). Many researchers have investigated the effect of inhibiting myostatin to counteract sarcopenia using animals (Siriett et al., 2006; LeBrasseur et al., 2009; Murphy et al., 2010). LeBrasseur et al. (2009) reported several positive effects of 4 weeks of treatment with PF-354 (24 mg/Kg) in aged mice. They found that PF-354-treated mice exhibited significantly greater muscle mass (by 12%) probably due to decreased levels of phosphorylated Smad3 and MuRF-1 in muscle. More recently, Murphy et al. (2010) showed, by way of once-weekly injections, that a lower dose of PF-354 (10 mg/Kg) significantly increased the fiber cross-sectional area (by 12%) and *in situ* muscle force (by 35%) of aged mice (21-month-old).

However, the role of myostatin in driving sarcopenia is debated. There is indeed evidence that myostatin null mice, although they have a doubling of muscle mass, have reduced specific force and may be actually prone to sarcopenia, suggesting that the intrinsic capacity to generate force is perturbed in the absence of myostatin (Amthor et al., 2007; Gentry et al., 2011). In addition, a recent study in *Drosophila* on the myostatin/GDF11 homolog myoglianin indicates that, in the absence of changes in muscle mass, overexpression of myoglianin (*Drosophila* myostatin) in muscle extends lifespan and preserves muscle function at least in part by activating the stress-sensing kinase p38 MAPK, while myoglianin RNAi in muscle has converse effects (Demontis et al., 2014; Patel and Demontis, 2014).

In rodent muscle models, studies using sarcopenic muscles have yielded conflicting results (Haddad and Adams, 2006; Carlson et al., 2008; Bowser et al., 2013). Haddad and Adams (2006) showed lower expression of myostatin mRNA in aged (30-month-old) than in young (6-month-old) rats. Carlson et al. (2008) showed higher levels of TGF-β and Smad3 but not myostatin in sarcopenic muscles of mice. In humans, an early cross-sectional study of younger, middle-aged, and older men and women suggested that serum myostatin levels increase with advancing age, are highest in “physically frail” older women, and are inversely associated with skeletal muscle mass (Yarasheski et al., 2002). However, several subsequent reports on humans failed to show age-related differences in either circulating myostatin-immunoreactive protein or skeletal muscle myostatin mRNA levels (Welle et al., 2003; Ratkevicius et al., 2011). In contrast, Léger et al. (2008) found a significant elevation in myostatin mRNA and protein levels by 2- and 1.4-fold in young (20 ± 0.2 years) males compared with those in older (70 ± 0.3 years) ones. These disparate findings suggest that myostatin may not be a primary driver of sarcopenia, or may instead highlight the complexities related to myostatin and its measurements. As indicated by a recent review (White and LeBrasseur, 2014), three possible reasons for this exist. First, myostatin abundance may not reflect myostatin activity. Indeed, myostatin is generated as a precursor protein that requires proteolytic cleavage first to remove its signal peptide and then to liberate an N-terminal propeptide and a C-terminal fragment. The mature biologically active form of myostatin is only a disulfide-linked dimer of C-terminal fragments. Second, myostatin is further regulated by at least three interacting proteins, namely, GDF-associated serum protein-1 (GASP-1), follistatin, and follistatin-related gene (FLRG) (Lee, 2004). It is plausible that the abundance of these endogenous inhibitors of myostatin and/or the degree to which they interact with myostatin is independently affected by aging. Third, we may not detect the expression pattern of myostatin during sarcopenia because of very small changes of this molecule at only a limited position of an organelle (e.g., satellite cells), but not throughout muscle fibers. Indeed, a recent study revealed that muscle-derived stem cells from older male patients show a +65% higher level of myostatin expression than stem cells from younger patients (McKay et al., 2012). Although myostatin immunoreactivity on satellite cells gradually decreased the response to acute resistance exercise, old muscles possessed more abundant myostatin on satellite cells of type II fibers than young muscles postexercise. More descriptive study to investigate a detailed cellular localization of myostatin would detect such a limited but important adaptation of myostatin in sarcopenic muscle.

### Functional role of myostatin in dystrophic muscle

There have been several studies dealing with the adaptive changes in myostatin expression of muscular dystrophy. Using muscles from fetopsies, infants (aged 8–10 months), and symptomatic patients (aged 5–12 years) with DMD, Chen et al. (2005) performed mRNA profiling. They demonstrated no induction of myostatin mRNA at any stage of the disease determined in their study. Similarly, no induction of myostatin was also observed in DMD muscle by Castro-Gago et al. (2006). Zanotti et al. (2007) showed significant increases in myostatin transcript and protein levels in DMD myotube cultures *in vitro*. In contrast, a screen of 12,488 mRNAs in 16-week-old mouse mdx muscle showed a marked decrease (fourfold) in myostatin mRNA (Tseng et al., 2002). Similar down-regulation of myostatin mRNA was observed in mdx mice using suppression subtractive hybridization (Tkatchenko et al., 2000). Therefore, myostatin does not seem to modulate the atrophy and degeneration of skeletal muscle in DMD and mdx mice, since common adaptation of myostatin levels did not occur in these dystrophic muscles.

Many mutations in the caveolin-3 gene have been detected in autosomal dominant LGMD1C and autosomal dominant rippling muscle disease (AD-RMD) (Minetti et al., 1998; Betz et al., 2001). Immunoprecipitation and subsequent immunoblot analysis revealed that caveolin-3 associates with the type I myostatin receptor in COS-7 monkey kidney cells *in vitro* (Ohsawa et al., 2008). Intriguingly, caveolin-3 seems to suppress myostatin signaling by blocking the type I myostatin receptor. Therefore, caveolin-3-deficient mice showed hyperphosphorylation of an R-Smad of myostatin, Smad2, and significant up-regulation of a myostatin target gene, p21 (Ohsawa et al., 2006). In addition, severe muscle histopathology was occasionally observed in the proximal muscles of patients with LGMD2I, whereas distal muscles were always relatively spared. In these patients, the amount of myostatin protein was highly increased in severely affected muscles compared with that in mildly affected ones. Hauerslev et al. (2013) hypothesize that alterations in the protein turnover and myostatin levels could progressively impair the muscle mass maintenance and/or regeneration, resulting in gradual muscular atrophy in LGMD2I. However, comprehensive analysis using a larger sample size of LGMD2I patients is needed as the hypothesis was generated from a very small sample size (severe phenotype *n* = 1; mild phenotype *n* = 3). In contrast, our previous study found a marked increase in mature myostatin protein (26 kDa) in gastrocnemius and rectus femoris muscles of merosin-deficient congenital dy mice at 12 weeks of age (Sakuma et al., 2004). In addition, marked myostatin immunoreactivity was detected in the cytoplasm of myonuclei and/or satellite cells of dy mice compared with slight myostatin immunoreactivity at those sites in normal mice. Therefore, muscular dystrophy except for dystrophin deficiency induces the enhancement of myostatin-dependent signaling.

Many therapeutic approaches using myostatin attenuation have been conducted in muscular dystrophy. The use of neutralizing antibodies to myostatin improved muscle disorders in rodent models of DMD (mdx) and limb-girdle muscular dystrophy 2f (Sgcg−/−) (Bogdanovich et al., 2002; Bradley et al., 2008). Targeting of the C-terminal dimer by a neutralizing monoclonal antibody (JA16) resulted in increases in muscle mass and function in wild-type mice (Whittemore et al., 2003) and rescued the pathological phenotype in dystrophin-deficient mdx mice (Bogdanovich et al., 2002). The latter study was the first to provide evidence that blocking myostatin in dystrophic mice increased myofiber size and alleviated the symptoms of the disease, such as a decline in strength, the degeneration of fibers, and fibrosis. The inhibition of myostatin was also effective in alleviating the pathological phenotype of caveolin-3-deficient mice (a model of LGMD1C) (Ohsawa et al., 2006). In contrast, myostatin blockade did not attenuate the pathology in a mouse model of merosin-deficient muscular dystrophy.

**Figure 2:**
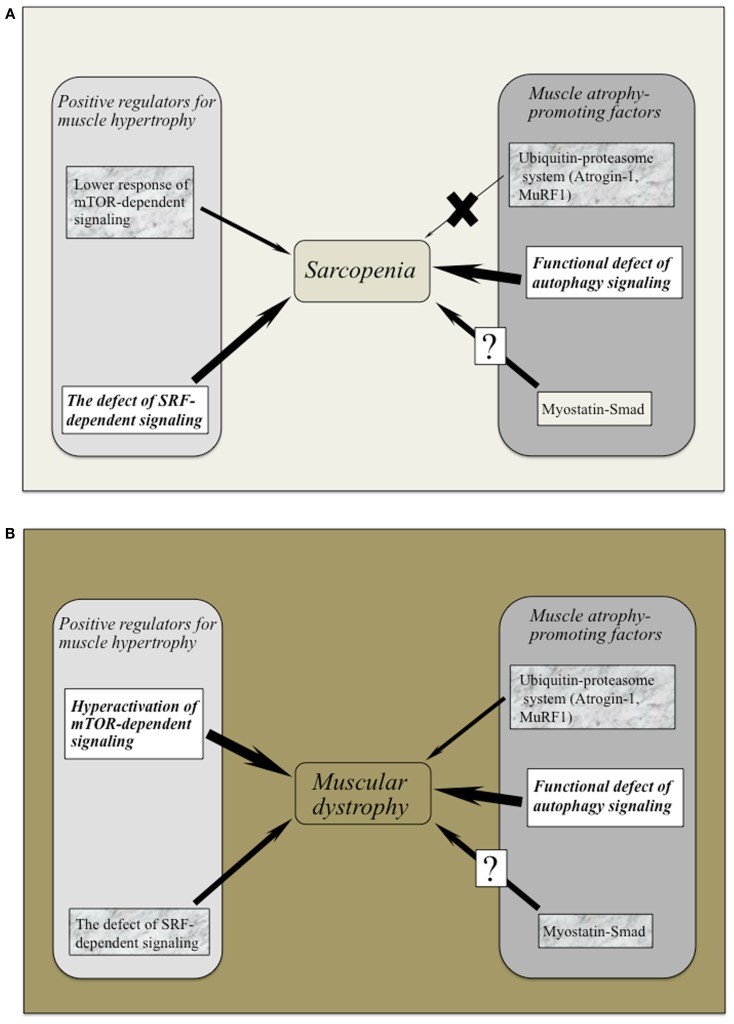
**(A,B)** The adaptative changes of positive and negative regulators for muscle mass in sarcopenia and muscular dystrophy. Both sarcopenia and muscular dystrophy exhibit the marked defect of autophagy-dependent signaling possibly the latter due to hyperactivation of Akt/mTOR/p70S6K pathway. Lower activation of SRF-dependent signaling has been commonly recognized in these symptoms. Ubiquitin–proteasome system (Atrogin-1 and MuRF-1) would not regulate muscle atrophy in the case of sarcopenia. It remains to be elucidated whether myostatin–Smad pathway regulates to sarcopenic symptom and/or muscular dystrophy.

Intriguingly, myostatin inhibition using MYO-029 (Stamulumab) was tested in a prospective, randomized, placebo-controlled US phase I/II trial in 116 adults with muscular dystrophy such as BMD, fascioscapulohumeral muscular dystrophy (FSHD), and LGMD (Wagner et al., 2008). MYO-029 has good safety and tolerability except for cutaneous hypersensitivity at higher doses (10 and 30 mg/Kg), attributed to the need for repeated protein administration (Wagner et al., 2008). No improvements in muscle function were noted, but dual-energy radiographic absorptiometry and muscle histological investigations revealed that some subjects had increased muscle fiber size. The trial study concluded that the systemic administration of myostatin inhibitors was relatively safe and that more potent inhibitors for stimulating muscle growth in muscular dystrophy should be considered. However, careful attention should be paid to myostatin inhibition, as mice with null mutation of myostatin revealed impaired tendon structure and function (Mendias et al., 2008). Figures 2A,B provide an overview of the positive and negative regulator adaptations of muscle mass in sarcopenia and muscular dystrophy.

## Conclusion

In conclusion, both sarcopenia and muscular dystrophy exhibit the marked defect of autophagy-dependent signaling possibly the latter due to hyperactivation of Akt/mTOR/p70S6K pathway. Lower activation of SRF-dependent signaling has been commonly recognized in these symptoms. Although studies using rodent muscles have indicated that Atrogin-1 and MuRF contribute to the protein degradation in muscular wasting (Bodine et al., 2001a), these atrogenes do not regulate age-related muscle atrophy. More descriptive study seems to have detected such a limited but important adaptation of myostatin during sarcopenia.

## Conflict of Interest Statement

The authors declare that the research was conducted in the absence of any commercial or financial relationships that could be construed as a potential conflict of interest.
